# Brain Metastasis Mimicking Glioma on Imaging Appearance During Tyrosine Kinase Inhibitor Administration: A Case Series and Literature Review

**DOI:** 10.7759/cureus.43591

**Published:** 2023-08-16

**Authors:** Yurie Rai, Hirokazu Takami, Kei Kawaguchi, Shunsaku Takayanagi, Shota Tanaka, Yoichi Yasunaga, Nobuhito Saito

**Affiliations:** 1 Department of Neurosurgery, The University of Tokyo Hospital, Tokyo, JPN; 2 Department of Pathology, The University of Tokyo Hospital, Tokyo, JPN

**Keywords:** non-small cell lung cancer, fdg-pet, mri, peritumoral edema, tyrosine kinase inhibitor, brain metastasis

## Abstract

Objective: Preoperative imaging diagnosis is critical to planning treatment strategies; however, it is occasionally challenging and sometimes misleading. The effects of molecularly targeted therapies on imaging appearances remain uncharted. We investigated the imaging characteristics of brain metastasis during tyrosine kinase inhibitor (TKI) administration.

Methods: We analyzed the 12 cases of brain metastasis from lung cancer in our institute, including a case of a 49-year-old woman under gefitinib. Additionally, we reviewed the cases of brain metastasis from lung cancer with gefitinib treatment in the literature.

Results: A woman during five-year gefitinib treatment for postoperative recurrence of lung adenocarcinoma was found to have a cerebellar tumoral lesion incidentally on magnetic resonance imaging (MRI). This lesion did not harbor any peritumoral edema, along with appearing hypometabolic on fluorodeoxyglucose (FDG) positron emission tomography (PET). This appearance was inconsistent with a typical metastatic appearance, and high-grade glioma was instead highly suspected, leading to a decision to proceed to gross total tumor resection. The pathological diagnosis, however, was brain metastasis from lung cancer. The other 11 cases without TKI treatment showed peritumoral edema on MRI and higher accumulation of FDG on PET. The two cases of brain metastasis with gefitinib in the literature showed no peritumoral edema on MRI.

Conclusion: TKIs like gefitinib can affect tumor biology, leading to a loss of typical imaging findings such as peritumoral brain edema and hyper-metabolism. As preoperative imaging diagnosis guides us in surgical planning, including biopsy or resection, ongoing treatment information should be fully integrated into imaging interpretation.

## Introduction

Exact imaging diagnosis for brain tumoral lesions by raising differential diagnoses and ranking them by likelihood carries significant clinical implications and is indispensable in formulating treatment strategies. For example, high-grade glioma (HGG) is known to be most effectively treated by safely maximizing tumor resection. Primary central nervous system lymphoma is now treated with methotrexate-based chemotherapy, with or without radiation therapy, saving surgery for tumor biopsy and pathological diagnosis. Brain metastasis generally warrants treatment by surgical resection when the lesion is large enough to cause neurological deficits due to mass effects, by radiation therapy including stereotactic radiosurgery, or by molecularly targeted therapy. With that mentioned, however, achieving the exact diagnosis based on imaging findings alone is sometimes challenging. Typical imaging findings for brain metastasis are contrast-enhanced lesions accompanied by prominent peritumoral brain edema (PTBE) and hypermetabolism represented on modalities such as 18F-fluorodeoxyglucose positron emission tomography (FDG-PET) [1]. HGG, in contrast, demonstrates varying degrees of PTBE and contrast enhancement, with various metabolic states on FDG-PET [2,3].

Here, we present a case of brain metastasis from non-small cell lung carcinoma (NSCLC) during gefitinib administration, with atypical imaging findings including a lack of peritumoral edema evident on magnetic resonance imaging (MRI) and hypometabolism on FDG-PET. A review of previous cases of brain metastasis from NSCLC in our institution and the existing literature corroborated the hypothesis that gefitinib administration might contribute to tumor quiescence leading to atypical imaging presentations. This would necessitate a guarded interpretation of imaging findings in diagnosis.

## Materials and methods

Case series

A total of 12 cases were identified in which resection pathologically confirmed brain metastasis from lung NSCLC during the period from 2014 to 2022 at The University of Tokyo Hospital, and for which preoperative images from both T2-weighted imaging (T2WI) and gadolinium-enhanced T1-weighted imaging (Gd-T1WI) were available (Cases #1-#12). For each case, the number of brain tumor lesions was counted, the diameter of the tumor was measured, and its associated PTBE was quantified. PTBE index was calculated as detailed in the Results section. Additionally, the tumor’s metabolic state was evaluated using FDG-PET. All pertinent components of this study were approved by our institutional review board, including the provision of an informed consent waiver for what was deemed a minimal-risk retrospective review. An illustrative case (Case #12) of brain metastasis with gefitinib administration is described in detail.

Literature review

To investigate the relationship between gefitinib administration and MRI, previous reports in the literature were identified and appraised. PubMed was queried using the key Medical Subject Headings (MeSH) terms of “brain metastasis”, “gefitinib”, “lung” and “MRI” for the period of January 2003 to October 2022. Initially, papers with full texts were included. Subsequently, in order to assess PTBE in a consistent manner, papers lacking images from T2WI or FLAIR were excluded. Then, full-length articles were reviewed, and cases with imaging performed during gefitinib administration with available images from T2WI or FLAIR were identified, and their PTBE was assessed.

## Results

Case illustration

A 49-year-old woman (Case #12) was brought to our attention for an investigation of an asymptomatic cerebellar hemispheric tumor. She had undergone initial surgical resection of lung adenocarcinoma nine years previously and second surgery for local recurrence five years previously. She had been taking gefitinib since that recurrence, for which she had achieved a complete response. A medical checkup detected a cerebellar lesion incidentally.

**Figure 1:**
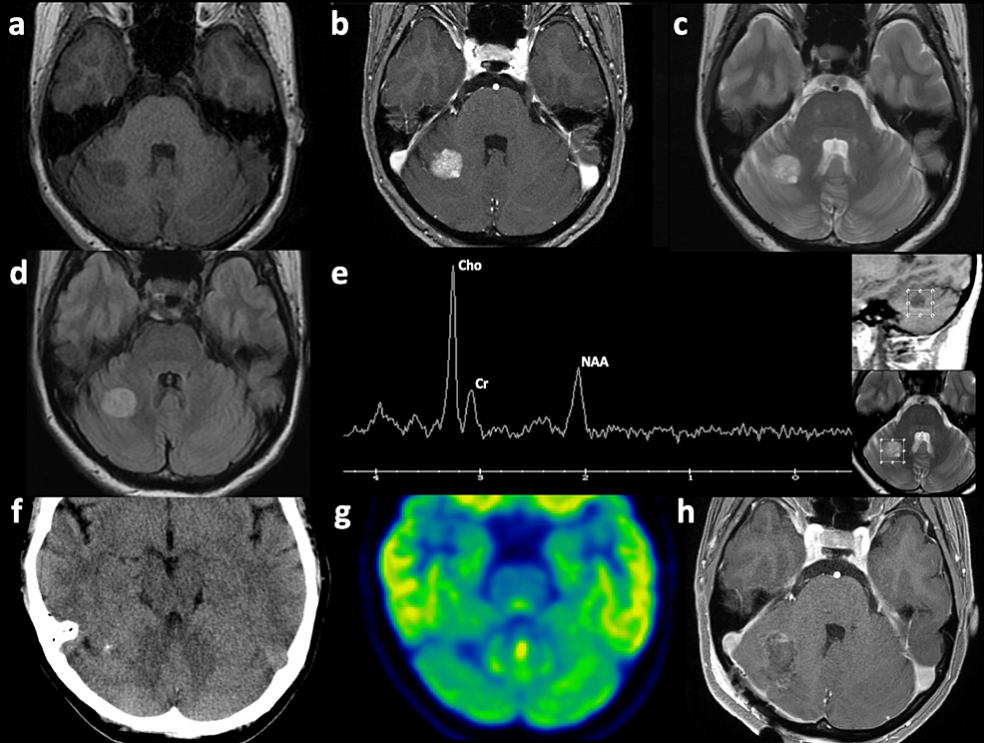
Pre- and postoperative images. T1WI of the brain shows a low-intensity intra-axial tumoral lesion in the right cerebellum (a), with heterogeneous gadolinium enhancement (b). T2WI (c) and FLAIR (d) show a heterogeneously hyperintense tumor without PTBE. (e) MRS shows peaks of Cho, Cr and NAA with a high ratio of Cho/NAA. (f) CT shows partial calcification in the tumor. (g) FDG-PET shows hypo-accumulation at the tumoral lesion with an L/N ratio of 0.50. (h) Postoperative gadolinium-enhanced T1WI of the brain shows gross total resection of the tumor.

Follow-up MRI showed an increase in size by 5 mm in the subsequent five months from the baseline size of 18 mm. The tumoral lesion appeared heterogeneously hyperintense on T2WI, and hypointense on T1WI, with heterogeneous gadolinium enhancement (Figures 1a-1d). No PTBE was observed on T2WI or fluid-attenuated inversion recovery (FLAIR) sequences (Figures 1c, 1d). Magnetic resonance spectroscopy (MRS) revealed peaks for choline (Cho), creatinine (Cr), and N-acetyl aspartate (NAA), with a high Cho-to-NAA ratio (Figure 1e). Computed tomography (CT) revealed partial calcification in the lesion (Figure 1f). FDG-PET demonstrated hypometabolism with a lesion-to-normal (L/N) ratio of 0.50 (Figure 1g). These imaging findings were congruent with cerebellar parenchymal tumor, most likely HGG. Clinically speaking, the previous lung cancer was well controlled and whole-body FDG-PET demonstrated no other findings of metastasis, supporting the interpretation that brain metastasis was less likely. Based on these thoughts, we performed a craniotomy and achieved gross total resection of the tumor (Figure 1h).

**Figure 2:**
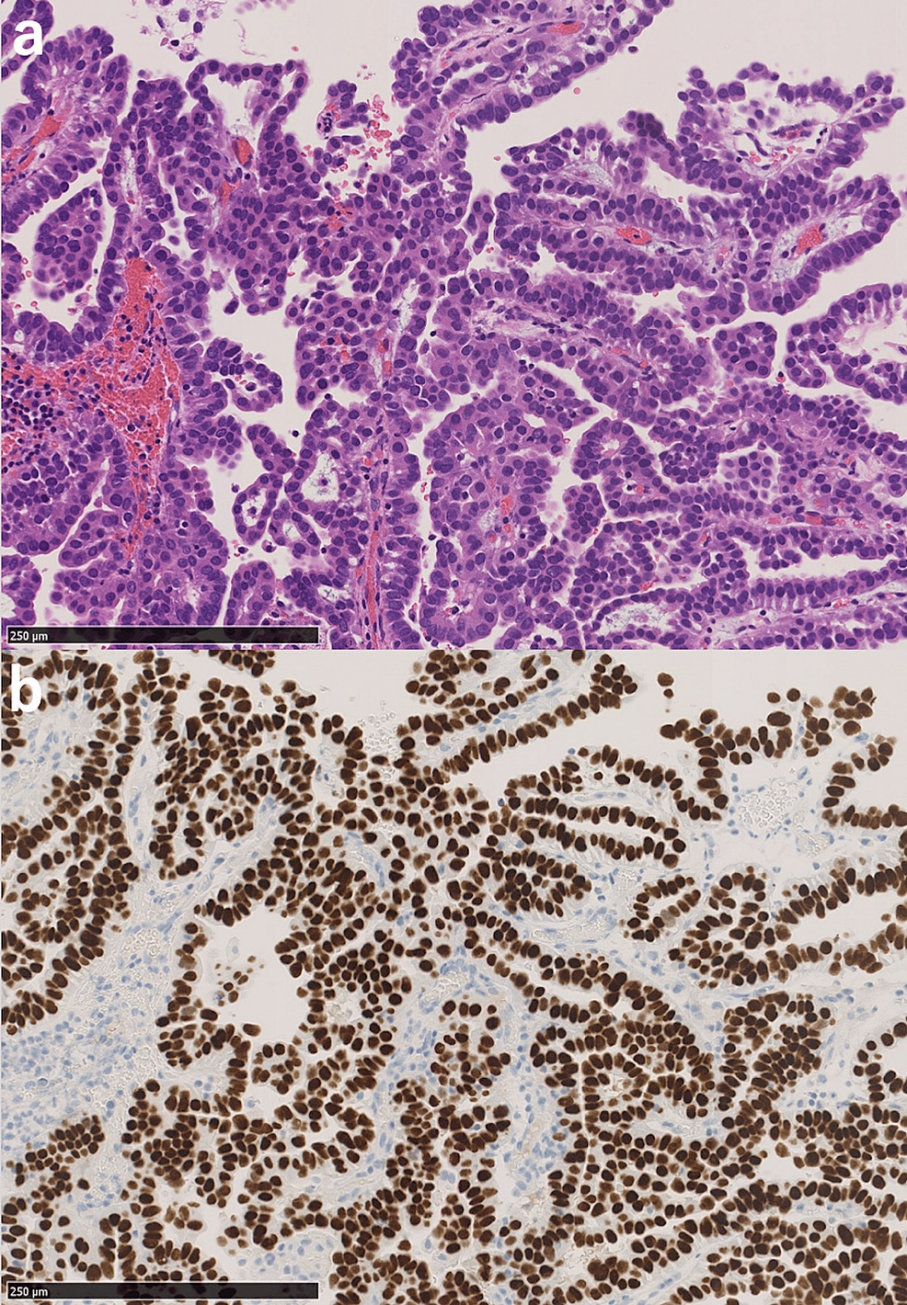
Pathological findings (scale bar, 250 µm). (a) Hematoxylin and eosin staining shows papillary growth of tumor cells with enlarged nuclei and eosinophilic cytoplasm. (b) Immunohistochemical imaging shows tumor cells are positive for thyroid transcription factor-1.

Pathological diagnosis of the resected specimen, however, showed adenocarcinoma. Immunohistochemical staining yielded positive results for thyroid transcription factor-1 (TTF-1) and napsin A and negative results for thyroglobulin, leading to the diagnosis of metastasis from lung adenocarcinoma (Figures 2a, 2b). Postoperatively, the patient recovered with no neurological deficits and was treated with stereotactic radiosurgery for the resection cavity.

Case series

Clinical information was collected using electronic charts, including the clinical characteristics of patients, previous treatments, and pathological findings (Table 1). Age ranged from 46 to 81 years and half of the patients were female (six cases). Ten cases were adenocarcinoma, one case was adenosquamous carcinoma and one case was NSCLC, not otherwise specified. Four cases showed EGFR mutation and only one of these patients (Case #12) was taking gefitinib at the time of imaging. Two cases (Cases #8 and #11) showed anaplastic lymphoma kinase (ALK) mutation and one had taken alectinib, an ALK inhibitor (Case #11).

**Table 1:** Brain metastasis from non-small cell lung cancer in our institute. F, female; M, male; CDDP, cisplatin; VNR, vinorelbine; PEM, pemetrexed; PTBE, petirumoral brain edema; BM, brain metastasis; ST, supratentorial; IT, infratentorial; BO, both; CR, complete response; SD, stable disease; PD, progressive disease; SYD, synchronous diagnosis of extracranial tumor and BM; GKS, gamma knife surgery

Case #	Age	Sex	Pathological diagnosis	EGFR mutation	ALK mutation	Preoperative therapy	KPS	Status of NSCLC	Steroid at imaging	MRI						FDG-PET L/N ratio
										PTBE	PTBE index	BM size (mm)	BM number	BM location	Hemorrhage	
1	75	F	Adenocarcinoma	L861Q	-	-	70	SYD	-	+	0.86	58	3	ST	-	1.31
2	55	M	NSCLC-not otherwise specified	-	-	-	90	SYD	-	+	1.19	32	1	ST	+	0.65
3	78	F	Adenocarcinoma	L858R	-	-	90	CR	-	+	3.71	17	1	ST	-	1.10
4	66	M	Adenocarcinoma	-	-	-	60	SYD	-	+	1.09	32	1	IT	-	0.63
5	72	M	Adenocarcinoma	-	-	-	80	SYD	-	+	0.72	54	1	ST	+	1.91
6	50	F	Adenocarcinoma	-	-	-	90	SYD	-	+	0.40	47	1	ST	+	1.06
7	81	F	Adenocarcinoma	L858R	-	-	80	SD	+	+	0.69	42	1	ST	+	1.80
8	68	M	Adenosquamous carcinoma	-	+	Imatinib, sunitinib, regorafenib	50	PD	-	+	1.00	21	1	ST	-	-
9	74	M	Adenocarcinoma	-	-	CDDP, VNR, PEM, GKS	70	SD	+	+	5.21	14	3	BO	-	1.29
10	71	F	Adenocarcinoma	-	-	CDDP, PEM	90	SD	-	+	0.40	20	1	ST	-	0.65
11	46	M	Adenocarcinoma	-	+	Alectinib, GKS	80	PD	+	+	1.00	42	1	ST	-	-
12	49	F	Adenocarcinoma	L858R	-	Gefitinib	100	CR	-	-	0.00	18	1	IT	-	0.50

For all 12 cases, MRI and FDG-PET findings were investigated. The number of brain tumor lesions was counted, and the maximum diameter of the largest tumor (a) was measured on Gd-T1WI in 10 cases, and on T2WI for the rest (Cases #7 and #8). Maximum diameter including any associated PTBE (b) was also measured at the same level in the corresponding image from T2WI. PTBE size was calculated as (b-a) and the PTBE index was defined as (b-a)/a [4]. Tumor size ranged from 14 to 58 mm (median, 32 mm). Except for the present case, PTBE was identified in all cases, ranging from 8 mm to 73 mm (median, 38 mm). PTBE index was from 0.40 to 5.21 (median, 1). FDG-PET was conducted in ten cases (except Cases #8 and #11) and the L/N ratio ranged from 0.50 to 1.91 (median 1.1), with the lowest L/N ratio of 0.50 found in the present case (Case #12). Among the four hypometabolic cases with L/N ratio < 1.00, two cases (Cases #2 and #4) showed sporadic hyper-accumulation inside the tumor lesion, and the other two cases (Cases #10 and #12) showed homogeneous hypo-accumulation throughout the entire tumor.

Collectively, although baseline characteristics and pathological findings for the patient did not appear atypical for the present case (Case #12) among the entire cohort (Cases #1-#12), imaging features were outstanding with a lack of PTBE and hypo-metabolic state. These findings provoked the idea that gefitinib administration might have contributed to the unusual imaging findings.

Literature review

A total of 43 articles were identified, three of which were excluded due to the unavailability of the full text. All figures in the articles were reviewed and articles without images from T2WI or FLAIR were excluded (n=35) owing to the difficulty of assessing PTBE. Full-length articles were reviewed (n=5) and two cases with imaging performed during gefitinib administration with images available from T2WI or FLAIR were finally identified.

Of particular note, the images in both cases showed no PTBE [5,6]. One case involved a 75-year-old man who had been taking gefitinib for 11 months for postoperative recurrence of lung adenocarcinoma. T2WI showed no PTBE [5]. The other case involved a 52-year-old woman. Following surgery, adjuvant chemotherapy, and radiotherapy for lung squamous cell carcinoma, an MRI of the brain showed multiple brain tumors with vasogenic edema. Repeat MRI after one month of gefitinib treatment showed a significant reduction in size with complete resolution of the associated vasogenic edema [6]. These findings were consistent with the hypothesis that gefitinib might be associated with a lack of PTBE. No relevant information regarding brain FDG-PET during gefitinib treatment was retrieved from PubMed.

## Discussion

MRI of brain metastasis most typically shows accompanying PTBE. Previous studies have analyzed PTBE for brain metastasis from NSCLC with respect to symptomatic/asymptomatic status [1] and mutational status [4], but not to medication including gefitinib. Only one case report on the relationship between PTBE and erlotinib appears to have been published. Essenmacher et al. reported a case of brain metastasis from lung adenocarcinoma treated with erlotinib who developed multiple cystic brain lesions lacking surrounding edema [7]. They suggested that this unique imaging finding might be related to the effects of erlotinib on metastatic brain tumors. That case differed from our present Case #12 in that the tumor itself showed a cystic appearance with no contrast enhancement. However, the cases were similar in that both had been treated with EGFR-TKI and showed no PTBE.

Of interest, one study evaluated the expression of hypoxia-induced factor 1 alpha (HIF1a) in brain metastasis [8]. That investigation found a significant correlation between small brain edema, low neo-angiogenic activity, and low expression of HIF1a. The EGFR-HIF1a signaling pathway is known to promote angiogenesis and invasion, leading to enhanced tumor activity in NSCLC [9,10]. Collectively, we assume that inhibition of the EGFR-HIF1a signaling pathway by EGFR-TKI administration suppressed the expression of HIF1a in the tumor, resulting in lower angiogenetic activity and leading to the lack of PTBE on imaging.

FDG-PET is feasible for diagnosing lung cancer metastasis with a sensitivity of 80%-100% and specificity of 93%-100%, although this modality is not routinely applied for assessing brain metastasis [11-13]. One report found that 31 of 42 cases of brain metastasis from NSCLC showed hypermetabolism on FDG-PET [14]. In our case series, eight of 11 cases without gefitinib showed hypermetabolism (L/N ratio > 1.0). Our Case #12 with gefitinib showed the lowest metabolism. In addition, several reports have shown that EGFR-TKI medication alone led to the regression of brain metastasis [6,15,16]. Correspondingly, we consider that suppression of tumor cell metabolism by EGFR-TKI treatment potentially results in a low accumulation of FDG-PET. These findings, including the loss of PTBE and low metabolism in relation to the intake of EGFR-TKI, need to be verified in a larger cohort clinically and ideally warrant biological investigations, for these observations to be considered clinically actionable information.

Retrospectively speaking for the present case, if we had suspected brain metastasis preoperatively, we could have planned biopsy alone to establish the diagnosis, then proceeded to stereotactic radiosurgery, which would have been less invasive for the patient. This case provides a good lesson that gefitinib might produce a glioma-mimicking appearance in brain metastasis.

## Conclusions

We report a case of NSCLC brain metastasis during gefitinib administration presenting imaging findings including a lack of PTBE and hypo-accumulation on FDG-PET, leading to misdiagnosis of HGG. We posit that these atypical imaging features and their background biology might reflect the effects of EGFR-TKI administration. Clinical information including current medication profiles should be fully integrated to achieve the most appropriate imaging diagnosis for brain tumors.
